# Recurrent stroke in a reproductive age women with patent foramen ovale

**DOI:** 10.1186/s12905-024-02909-3

**Published:** 2024-01-25

**Authors:** Jing Ye, Yujia Yang, Li Tang, Li He, Muke Zhou

**Affiliations:** Department of Neurology, West China Hospital, Sichuan University, Chengdu, China

**Keywords:** Stroke, Patent foramen ovale, Combined oral contraceptives, Women

## Abstract

**Background:**

Patent foramen ovale (PFO) is a known cause of ischemic stroke in young adults and combined oral contraceptives (COCs) are widely used by women of reproductive age. If young women with PFO are taking COCs, they may be subjected to a synergistic increase in the occurrence of stroke, though reports of ischemic stroke in this population are rare. We report a woman of reproductive age who was taking COC suffered repetitive ischemic strokes before a patent foramen ovale (PFO) was detected and closed, which may raise concerns in this field.

**Case presentation:**

A 31-year-old woman presented to the emergency department with sudden-onset right upper- and lower-limb weakness and dysarthria for 1 hour, whose only risk factor of stroke was oral contraceptive use. On admission, she was alert with left gaze deviation, dysarthria, and right-sided hemiplegia. Her symptoms improved after receiving the revascularization therapy. About 24 hours later, her left eye experienced sudden painless vision loss. Then the PFO with a substantial right-to-left shunt was detected and then she received a trans-catheter closure of the defect. Over 3 months of follow-up, there were no signs of stroke, but visual loss persisted.

**Conclusion:**

This case of disabling stroke raises concerns regarding optimal management in primary and secondary prevention of stroke in young women on COCs with additional risk factors of stroke.

## Background

Patent foramen ovale (PFO), which occurs in nearly 25% of healthy individuals, is the most common cardiac finding in young adults who suffer a cryptogenic stroke episode [1]. It has been suggested that a PFO could facilitate thrombus formation or serve as a conduit for paradoxical embolism [1]. Women of reproductive age are often prescribed combined oral contraceptives (COCs), which have been shown to increase the risk of arterial and venous thromboembolic events [2]. If young women with PFO take COCs, they may be subjected to a synergistic increase in risk of stroke [3] though reports of ischemic stroke in this population are rare [4].

Here we present the case of a young woman who was on a COC and suffered repetitive ischemic strokes before a PFO was detected and closed. This case of disabling stroke raises concerns regarding optimal management in primary and secondary prevention of stroke in women [4].

## Case presentation

**Fig. 1:**
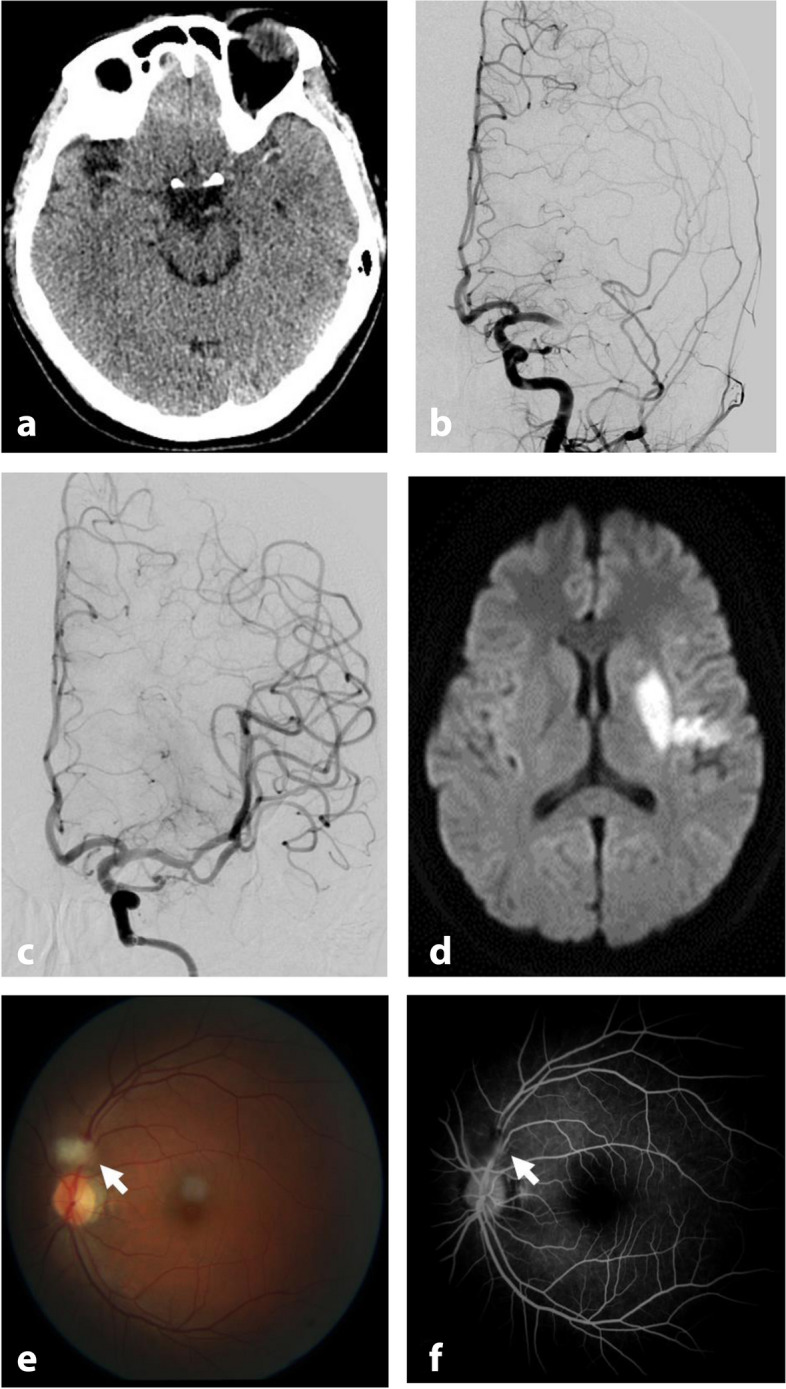
Imaging of vessel occlusion and resolution. **a** Head computed tomography image showing a hyperdense left middle cerebral artery (MCA) sign. **b** Diagnostic cerebral angiography demonstrating occlusion of the left MCA M1 segment. **c** Postoperative cerebral angiography demonstrating reperfusion of the MCA following mechanical thrombectomy. **d** Diffusion-weighted brain MRI demonstrating lesions in the left basal ganglia. **e** and **f** Fundus fluorescein angiography images demonstrating obstructed branches of left retinal artery

A 31-year-old woman presented with sudden-onset right upper- and lower-limb weakness and dysarthria for 1 hour. She denied any history of hypertension, diabetes, heart disease, migraine or other cerebrovascular risk factors, a family history of stroke, previous trauma, smoking or toxin exposure. However, she said she had taken combined oral contraceptive (30 μg ethinylestradiol and 3 mg drospirenone) once a day for 30 days. On admission, her vital signs were normal. The findings of her general physical examination were normal. Her neurological examination revealed that she was alert with left gaze deviation, dysarthria, and right-sided hemiplegia. The patient’s National Institutes of Health stroke score (NIHSS) was 11. Her routine blood test, glucose levels, and electrocardiography results were normal. A head computed tomography showed a hyper-dense left middle cerebral artery (MCA) sign (Fig. 1A), prompting a diagnosis of acute ischemic stroke. She was given intravenous thrombolysis with alteplase (a recombinant tissue plasminogen activator), an acute cerebral angiography confirmed occlusion of the left MCA M1 segment (Fig. 1B), and then she received mechanical thrombectomy that achieved grade-3 reperfusion on the Thrombolysis in Cerebral Infarction scale (Fig. 1C). After revascularization, all of her symptoms except for the facial paralysis improved. Subsequent diffusion-weighted brain MRI demonstrated lesions in the left basal ganglia consistent with cerebral infarction (Fig. 1D). After about 24 hours, she developed sudden painless vision loss in her left eye. Fundus fluorescein angiography revealed obstructed branches of left retinal artery (Fig. 1E and F). She rejected ophthalmologist-recommended dexamethasone treatment.

**Fig. 2:**
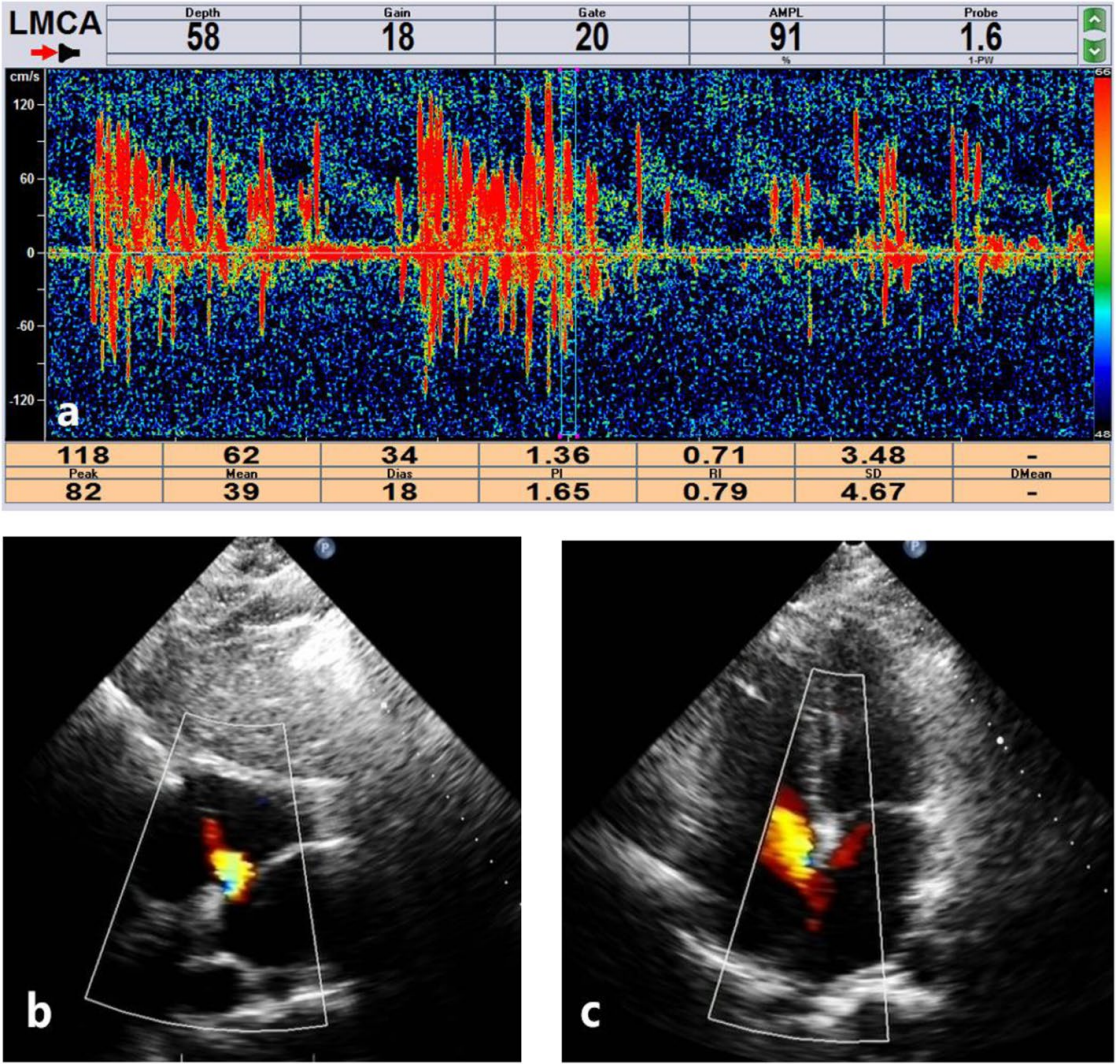
**a **Transcranial Doppler (TCD) result showing high-intensity transient signals, several of which masked background waveforms. **b** and **c** Transthoracic echocardiography (TTE) images of patent foramen ovale (PFO)

Her urine test, lipid panel, hemoglobin A1c measurement, liver, kidney and thyroid function, coagulation function, protein S, protein C, anti-cardiolipin antibody, anti-neutrophil cytoplasmic antibody, thrombophilia screening, C-reactive protein, HIV and syphilis serology, tumor markers, carotid duplex ultrasonography, transthoracic echocardiography (TTE) and 24-hour electrocardiogram were negative and unremarkable. Contrast-enhanced transcranial Doppler (TCD) analysis (Fig. 2A) revealed a sprinkling of microbubbles both at rest and after the Valsalva maneuver suggestive of a substantial right-to-left shunt (RLS). The patient refused transesophageal echocardiography (TEE). Repeated TTE demonstrated a PFO with interatrial RLS even without the Valsalva maneuver (Fig. 2B and C). Her pelvic, upper-limb, and lower-limb duplex venous ultrasound findings were unremarkable. She was prescribed a prophylactic oral blood thinner (oral clopidogrel, 75 mg/once daily). Her RoPE (Risk of Paradoxical Embolism) score was 9, indicating that there was a high probability that the detected PFO was causally related to her strokes. Based on the substantial RLS and high RoPE score, a trans-catheter closure of the defect was performed with an Amplatzer septal occluder (15 mm) 2 weeks later [5].

Over the subsequent 3 months of follow-up, there were no signs of stroke or transient ischemia attacks, but visual loss in the patient’s left eye persisted. Her best corrected visual acuity was 1.0 in her right eye and 0.5 in her left eye. Repeat contrast-enhanced echocardiography revealed a residual RLS evidenced by > 10 bubbles in the left atrium in the third cardiac cycle following contrast appearance in the right atrium after Valsalva release.

## Discussion and conclusion

PFO is a common etiology of cryptogenic stroke in young adults and COCs are widely used by young women.

Oral contraceptive use and RLS presence are considered decisive risk factors in otherwise healthy young stroke patients [6]. Most gynecological diseases require COCs and the population we care about is also this group of women who have to take oral contraceptives for a long time especially if they also have risk factors. If women show additional vascular risk factors, we recommend other contraceptive methods than oral contraceptives, such as condoms. COCs users who have additional risk factors, such as smoking, hypertension, and migraine with aura, are at an elevated risk of ischemic stroke [3, 7]. Similarly, women with a PFO who are taking COCs may have a higher occurrence of ischemic stroke than women with a PFO who are not taking COCs or women without an RLS taking COCs. Theoretically, there could be synergism of PFO and COCs use for ischemic stroke.

The present case raised two notable concerns. Firstly, PFO is not considered a contraindication for COCs use. Screening for the presence of RLS and other conventional risk factors prior to prescription of COCs may be necessary and reasonable for young women, particularly for those patients with a PFO-associated condition, such as migraine. Individuals who have the two risk factors of migraine and COCs use have been reported to have an odds ratio for ischemic stroke in the range of 5–17 [3], though PFO could be a confounding factor in the risk analyses. COCs users should be assessed regularly for the development of additional risk factors. Whenever possible, emerging risk factors should be modified before an affected patient continues or returns to using a COC. However, factors that cannot be controlled easily, such as migraine or PFO, represent a challenging problem in patients in whom a COC is indicated. Conversely, COCs should be prescribed with caution in women in whom a PFO has been identified. Neurologists and gynecologists should inform such patients that they are at increased risk of ischemic stroke, prescribe low-estrogen COCs for them if possible [3], provide intensive education on the identification of early signs of an ischemic event, and screen for and modify inasmuch as it is possible other potential risk factors [3]. Because few stroke cases and studies involving young women with a PFO taking COCs have been reported, there are insufficient data to determine a standard of care that can minimize stroke risk for patients in this population with or without a history of ischemic events.

PFOs can be detected by TTE, TEE or TCD. Of these methods, TEE provides the greatest sensitivity and specificity for PFO diagnosis and PFO morphology characterization, but it also is the most invasive. When TEE is not tolerated by patients and a significant RLS has been found by contrast-enhanced TCD, repetitive TTE and intra-operative intra-cardiac echocardiography can be performed to evaluate PFO closure.

Although the limb and pelvic duplex venous ultrasound findings were unremarkable in the present case, the thrombus was presumed to be of venous origin because systemic venous embolism is common in adults, is typically characterized by silent superficial venous thrombus formation, and can generate numerous migrating emboli [1, 8]. Additionally, the patient was taking a COC, which was an overt risk factor for thromboembolism.

Residual shunting is observed in about one in four patients after PFO closure and can leave patients, and especially those with a moderate or large residual shunt may cause stroke or transient ischemic attacks. Therefore, it is important to provide such patients with long-term care involving a multi-disciplinary team and with particularly close monitoring during the first year postoperatively because residual shunts tend to diminish with ongoing epithelialization and shunt stabilization.

## Take-home points

- In young women taking COCs with PFO or additional risk factors for stroke there may be a synergistic risk increase for ischemic events.
- Routine RLS screening could be considered in women of reproductive-age prior to COCs use.
- Because COCs risk is related to estrogen exposure, low-dose estrogen COCs should be prescribed if possible when COCs use is needed in women with a PFO.
- Evidence is lacking for effective primary and secondary prevention of stroke in COC users with a PFO.

## Data Availability

Not applicable.

## Abbreviations

PFO: Patent foramen ovale
COC: Combined oral contraceptive
NIHSS: National Institutes of Health stroke score
MCA: Middle cerebral artery
TTE: Transthoracic echocardiography
TCD: Transcranial Doppler
RLS: Right-to-left shunt
TEE: Transesophageal echocardiography
RoPE: Risk of Paradoxical Embolism
